# ARCII: A phase II trial of the HIV protease inhibitor Nelfinavir in combination with chemoradiation for locally advanced inoperable pancreatic cancer

**DOI:** 10.1016/j.radonc.2016.03.021

**Published:** 2016-05

**Authors:** James M. Wilson, Emmanouil Fokas, Susan J. Dutton, Neel Patel, Maria A. Hawkins, Cynthia Eccles, Kwun-Ye Chu, Lisa Durrant, Aswin G. Abraham, Mike Partridge, Martha Woodward, Eric O’Neill, Tim Maughan, W. Gillies McKenna, Somnath Mukherjee, Thomas B. Brunner

**Affiliations:** a Department of Oncology, CRUK/MRC Institute for Radiation Oncology, University of Oxford, UK; b Centre for Statistics in Medicine, Nuffield Department of Orthopaedics, Rheumatology and Musculoskeletal Sciences, University of Oxford, UK; c Department of Radiology, Oxford University Hospitals NHS Foundation Trust, UK; d Department of Radiotherapy, Oxford University Hospitals NHS Foundation Trust, UK; e Early Phase Research Hub, Department of Oncology, Oxford Cancer and Haematology Centre, Oxford University Hospitals NHS Foundation Trust, UK; f Department of Radiation Oncology, University of Freiburg, Germany; g German Cancer Consortium (DKTK), Heidelberg, Partner Site Freiburg, Germany

**Keywords:** Nelfinavir, Trial, Pancreatic cancer, Hypoxia, Imaging, Radiosensitisation

## Abstract

**Background and purpose:**

Nelfinavir can enhance intrinsic radiosensitivity, reduce hypoxia and improve vascularity. We conducted a phase II trial combining nelfinavir with chemoradiotherapy (CRT) for locally advanced inoperable pancreatic cancer (LAPC).

**Materials and methods:**

Radiotherapy (50.4 Gy/28 fractions; boost to 59.4 Gy/33 fractions) was administered with weekly gemcitabine and cisplatin. Nelfinavir started 3–10 days before and was continued during CRT. The primary end-point was 1-year overall survival (OS). Secondary end-points included histological downstaging, radiological response, 1-year progression free survival (PFS), overall survival (OS) and treatment toxicity. An imaging sub-study (*n* = 6) evaluated hypoxia (^18^F-Fluoromisonidazole-PET) and perfusion (perfusion CT) during induction nelfinavir.

**Results:**

The study closed after recruiting 23 patients, due to non-availability of Nelfinavir in Europe. The 1-year OS was 73.4% (90% CI: 54.5–85.5%) and median OS was 17.4 months (90% CI: 12.8–18.8). The 1-year PFS was 21.8% (90% CI: 8.9–38.3%) and median PFS was 5.5 months (90% CI: 4.1–8.3). All patients experienced Grade 3/4 toxicity, but many were asymptomatic laboratory abnormalities. Four of 6 patients on the imaging sub-study demonstrated reduced hypoxia and increased perfusion post-nelfinavir.

**Conclusions:**

CRT combined with nelfinavir showed acceptable toxicity and promising survival in pancreatic cancer.

Pancreatic ductal adenocarcinoma is one of the most aggressive cancers, and surgical resection is the only potentially curative option [1]. However, the majority of patients are diagnosed in late stages. Locally advanced pancreatic cancer (LAPC) is associated with a poor prognosis [2]. Definitive chemo-radiotherapy (dCRT) is considered a treatment option LAPC, however the overall survival (OS) with upfront dCRT ranges from 7–12 months [3], [4]. Pancreatic tumours are inherently resistant to radiotherapy, and dose escalation is limited by potential toxicity from irradiation of surrounding organs at risk (OARs), most commonly the gastro-intestinal tract [5]. Hypoxia and hypovascularity contribute to radioresistance in pancreatic cancer [6], [7], [8], [9]. We have previously demonstrated that the anti-retroviral drug Nelfinavir can enhance intrinsic radiosensitivity, reduce hypoxia and improve vascularity, potentially through Akt inhibition [10], [11], [12].

A phase I study (ARC I) combining nelfinavir with gemcitabine and cisplatin-based CRT in LAPC, demonstrated acceptable toxicity [13]. We therefore designed a single arm phase II trial to assess the efficacy and tolerability of this regimen. We hypothesised that the addition of Nelfinavir to CRT would improve OS with acceptable toxicity. In order to objectively demonstrate changes in hypoxia and perfusion due to Nelfinavir treatment, an imaging sub-study was included.

## Methods

### Study design and patients

ARC II was a single-centre, open label, phase 2 trial. Patients aged 18 years or older were eligible if they had histology or cytology proven pancreatic, ampullary or intra-pancreatic bile duct adenocarcinoma where the disease was locally advanced, inoperable and non-metastatic (or medically inoperable due to patient co-morbidity). Patients were required to have Karnofsky performance index ⩾70%, adequate liver, renal and bone marrow function, and estimated life expectancy ⩾12 weeks. All patients were staged with FDG PET-CT and discussed at the pancreatic multi-disciplinary meeting (MDM) where specialist pancreatic surgeons, radiologists and oncologists agreed on decision regarding inoperability and suitability for CRT. The local research ethics committee approved the study. All patients provided written informed consent. The ARC II trial was funded and sponsored by the University of Oxford. In June 2013 the protocol was amended to allow patients to enter a functional imaging sub-study (described below).

### Study procedures

**Fig. 1:**
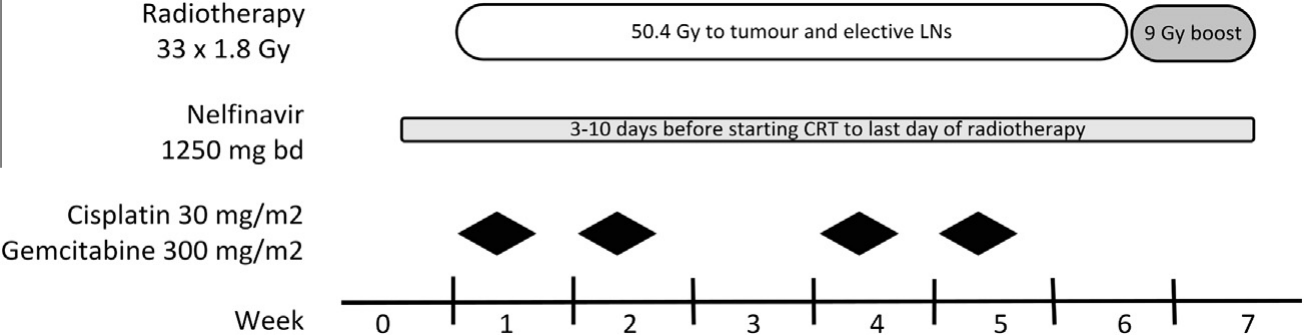
ARC-II trial design.

All patients received upfront CRT (Fig. 1). Patients underwent contrast-enhanced planning computer tomography (CT) simulation after a 2 h fast with 100–200 ml water as oral contrast. The gross tumour volume (GTV) with a margin of 2 cm cranio-caudal and 1.5 cm circumferential margin received 59.4 Gy in 33 daily fractions. Uninvolved loco-regional nodes received 50.4 Gy in 28 fractions. Daily online cone beam CT was used for treatment verification. Gemcitabine (1000 mg/m^2^) and Cisplatin (30 mg/m^2^) were given on weeks 1, 2, 4 and 5 during radiotherapy. Nelfinavir (1250 mg bd daily) was started 3 days (this was increased to 10 days following imaging amendment) prior to radiation, and continued until the last day of CRT. Following CRT all patients were evaluated for resectability. Adjuvant Gemcitabine for 6 months was recommended, but not mandated.

Treatment toxicity was assessed as per Common Terminology Criteria of Adverse Events (CTCAE version 4.0). Treatment compliance of Nelfinavir was assessed by tablet count. Clinic assessments including blood tests (haematological and biochemical tests, CA19.9) were performed at baseline, weekly during CRT, 6–8 weeks after CRT and 3 monthly thereafter until 12 months after trial entry. CT scan was performed at baseline, 6–8 weeks after completion of CRT, and 3 monthly until 12 months or progression. PET scan was performed at baseline and 6–8 weeks after completion of CRT. Following treatment, all patients were discussed at the MDM for resectability. Treatment after progression and patient management beyond 12 months were as per investigator choice.

### Functional imaging sub-study

For the imaging sub-study, patients underwent 18F-Fluoromisonidazaole-PET/CT (FMISO-PET) and perfusion CT (pCT) 24–48 h prior to starting nelfinavir and on day 6 or 7 of nelfinavir treatment.

### Assessment of peripheral blood mononuclear cell (PMBC) Akt phosphorylation

Phosphorylated Akt (pAkt) is downstream target of nelfinavir. pAkt (Ser 437) expression was assessed in PBMC using western blot as previously described [13].

### End-points and statistical analysis

The primary end-point of the study was 1-year OS after trial entry. Secondary end-points included histopathological downstaging (in resected patients), RECIST-based response (CT scan) and FDG PET-based response to therapy, 1-year progression free survival (PFS), OS, site of treatment failure and treatment toxicity.

The sample size calculation was based on the proportion surviving to 1 year (it was not expected that any patients would be lost to follow-up). If this proportion was 55% or greater treatment would be considered promising; if 40% or less, it would not be investigated further. Based on the above parameters and using a power of 80% and a significance level of 10%, the Fleming design requires 49 patients to take part in the study. If 27 or more patients are alive at 1 year, this will be taken as a sign of promising activity worth further investigation.

All analysis was undertaken using Stata 13.1 (StataCorp LP, College Station, TX, USA). Descriptive statistics are presented with mean (SD) for continuous variables, and numbers and percentages for binary or categorical variables. Proportions of patients still alive at 1 year are reported with 90% confidence intervals and a significance level of 10% was used for the sample size calculation.

This trial was registered at European Clinical Trials Register (EudraCT Number 2008-006302-42).

## Results

### Follow-up and survival

**Fig. 2:**
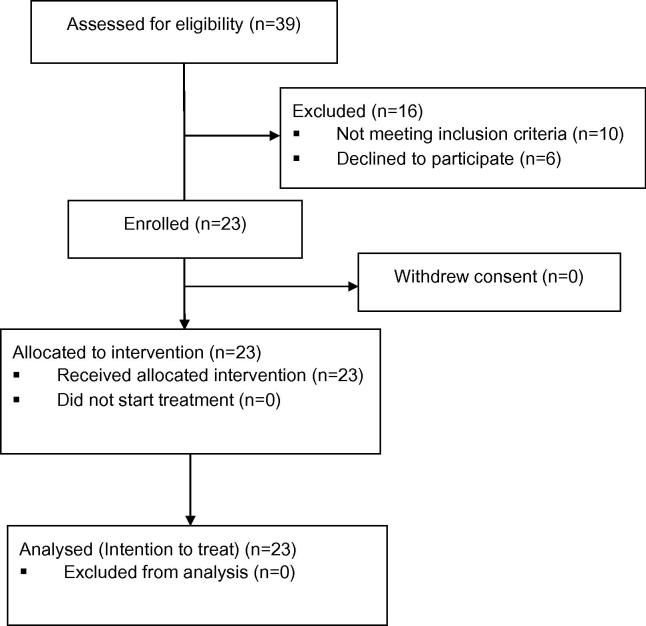
Consort diagram summarising the flow of patients.

Between January 2010 and July 2014, 23 patients were entered into the study. The study was discontinued in July 2014 following unavailability of Nelfinavir in Europe. The patient flow is shown in the CONSORT diagram (Fig. 2). Patients were followed until death, progression or 12-month assessment. Table 1 summarizes the baseline patient and tumour demographics. Median follow-up time was 14 months (IQR: 8.4–18.5) and all 23 patients were assessable for the primary end-point. Nineteen participants have died at time of analysis. Cause of death was related to pancreatic cancer in 17 participants; one participant died from a pulmonary embolism and in the remaining participants the cause of death was unknown.

**Fig. 3:**
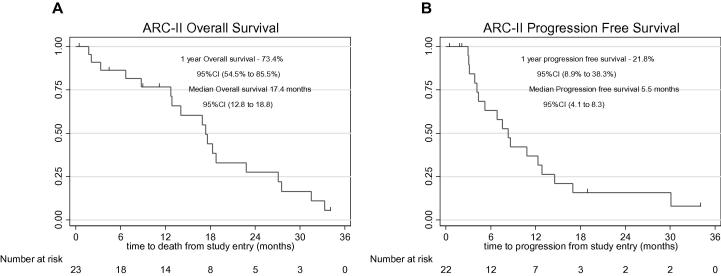
Clinical outcome in patients treated within the ARC-II clinical trial. Kaplan–Meier estimates of (A) Overall survival and (B) progression-free survival.

The 1-year OS after trial entry was 73.4% (90% CI: 54.5–85.5%). The median OS was 17.4 months (90% CI: 12.8–18.8). The corresponding Kaplan–Meier graph is displayed in Fig.3A. The 1-year PFS after trial entry was 21.8% (90% CI: 8.9–38.3%). The median PFS was 5.5 months (90% CI: 4.1–8.3). The corresponding Kaplan–Meier graph is displayed in Fig.3B.

Progression occurred in 19 participants (17 of whom died during follow-up). The site of first progression was local in 4 and distant in 15 participants. First sites of distant metastases were peritoneum (*n* = 5), liver (*n* = 5), lung (*n* = 3), both liver and lung (*n* = 1) or mediastinal node (*n* = 1).

Median follow-up time for patients with local progression was 18.3 months (IQR: 13.3–24.9). Nineteen of 23 patients were evaluable for local control at 1 year (2 died during follow up before 12 months; 1 did not complete treatment and 1 died at the end of treatment from pulmonary embolism). Twelve patients (12/19, 63.1%) had no evidence of local progression at 1 year. Additionally, neither of the two patients who died within 12 months of follow-up had evidence of local progression at the time of last CT scan.

The objective disease response by RECIST was: complete – nil; partial – 5 (21.7%); stable – 10 (43.5%); progression – 6 (26.1%). Two participants were non-evaluable – one had received less than 14 days of Nelfinavir (discontinued due to persistent hyperbilirubinaemia) and the other died due to a pulmonary embolism prior to end of treatment. Two patients (8.7%) underwent a resection, both had negative (R0) resection margins. The pathological stages of the two patients were pT0pN0M0, L0V0R0, Gx and pT3pN1M0, L1V0R0, G2, respectively. At the time of analysis, one resected patient was alive and progression free 13.9 months from study entry. The other patient relapsed with lung metastasis and survived 31.5 months from study entry.

### Toxicity and treatment tolerance

The main Grade 3–4 toxicities are outlined in Table 2. All patients experienced Grade 3/4 toxicity. Asymptomatic Grade 3–4 metabolic (laboratory) adverse events were seen in 10 (43.5%) patients. Grade 3–4 lymphopenia, a well-recognised feature of Nelfinavir, was seen in 20 (87%) patients but did not require supportive treatment. Common Grade 3/4 non-haematological toxicities were diarrhoea (*n* = 5, 21.7%), nausea/vomiting (*n* = 5, 21.7%), fatigue (*n* = 4, 17.4%) and infection (*n* = 3, 13%). One patient died during the final week of treatment from pulmonary embolism.

Supplementary Table 1 shows treatment tolerance during CRT. Radiotherapy was well tolerated, with 73.9% of patients receiving full dose radiation and 87% of patients receiving at least 54 Gy. Similarly at least 87% of the participants received 80% or more dose of Nelfinavir. Concomitant chemotherapy was less well tolerated but 78.3% of participants still received at least 80% of the protocol dose.

### Functional imaging, CA19.9 response and phospho-AKT assessment

#### FDG-PET

Median FDG SUV_max_ pre- and post-CRT was 7.6 (range 2.6–15.6) and 3.8 (1.6–7.9) respectively (*p* = 0.002). The median change (%Δ) in FDG SUV_max_ was −44.2%. Patients with %Δ SUV_max_ > median had a median OS of 23.0 months compared to 14.6 months with %Δ SUV_max_ < median (*p* = 0.01).

#### FMISO-PET and perfusion CT

Four of 6 patients recruited to this sub-study had reduced f-MISO retention with corresponding increase in pCT derived blood flow (BF) post-nelfinavir. Mean change in f-MISO-k3 (2 tissue compartmental model) – was 50.3% vs 6% and BF 20.1 vs −7.1% in responders vs non-responders.

#### CA19.9

The median CA19.9 level at baseline was 387 U/ml (IQR: 68–1711). At week 13, this dropped to 122 U/ml (IQR: 27–410). The median change in CA19.9 from baseline to week 13 was −429 U/ml (IQR: −1992 to −19). A decline in CA19.9 was not predictive of overall or progression free survival.

#### Phospho-AKT assessment

Serial blood measurement of phospho-AKT in PBMCs was available in 13 patients. Eight of 13 patients demonstrated a reduction in pAKT 7 days after initiation of treatment, consistent with Nelfinavir effect.

### Post-CRT chemotherapy and 2nd line treatment

Following CRT, six patients received adjuvant Gemcitabine, two of whom progressed during treatment. Ten patients received second-line treatment on progression, of whom 4 received gemcitabine and 6 patients received non-gemcitabine regimen [oxaliplatin–capecitabine (*n* = 3), FOLFIRINOX (*n* = 2), mitomycin (n = 1)].

## Discussion

The present phase II study reports the clinical outcome in 23 patients with LAPC treated with CRT plus nelfinavir. The median and 1-year OS were 17.4 months and 73.4%, respectively. Failure at first relapse was local in 4 patients only with a 1-year local control rate of 63.1%. Although incidence of Grade 3/4 toxicity was high, 87% of patients received 90% radiation dose; 78.3% and 87% of patients received at least 80% dose of chemotherapy and nelfinavir respectively, suggesting that the toxicity was manageable.

The prognosis for patients with LAPC is poor [1] and the role of RT in this disease remains controversial, particularly due to the high incidence of early metastatic spread [3], [4], [14]. However, 30% of LAPC never develop metastases [15] and local tumour progression represents a significant cause of disease-related morbidity and mortality [16], [17]. In ARCII, the predominance of systemic failures despite patient selection though PET-CT suggests that in addition to imaging, appropriate molecular markers of early metastasis like SMAD4, p53 [15], [18] needs to be investigated to select patients who are most likely to benefit from radiation.

Previous studies assessing efficacy of upfront CRT has reported OS outcomes of approximately 7–12 months [19], [20], [21], [22], [23] and inadequate adjuvant chemotherapy has been implicated in the poor survival seen in a previous dCRT trial [30]. More recently there has been a shift in practice to using 3–4 months of induction chemotherapy to select patients for CRT, as this has demonstrated OS rates of approximately 11–19 months [24], [25], [26], [27], [28], [29]. The median OS of 17.4 months observed in ARCII is superior to historical survival rates seen after upfront dCT and suggests that in appropriately selected patients this could still be an option, and may be a useful approach particularly where local symptoms dominate or where downstaging to surgical resectability is still considered a possibility.

To date, only phase I trials of nelfinavir in combination with (chemo)radiation have been conducted in different tumour types including pancreas [13], [31], [32], [33], [34]. In these studies Grade 3–4 toxicity varied between 17% and 46%. The toxicity profile in ARC II was consistent with that seen in the phase I pancreatic study [13] but higher than reported in recent pancreatic phase II–III CRT trials [24], [26]. However, the toxicity pattern suggests that this is more likely to have resulted from the concomitant Gem-Cis chemotherapy, higher RT dose and the large RT field rather than from nelfinavir itself [35].

Although initially designed as an inhibitor of the human immunodeficiency virus (HIV) protease, nelfinavir also inhibits Akt phosphorylation and activation [10], [36] that can radiosensitise tumours [10], [12], [37]. Notably, we have recently found that nelfinavir enhances the response of pancreatic cancer cells to radiotherapy in normoxic and hypoxic conditions, both in the absence and presence of pancreatic stellate cells (PSCs). In-vivo, administration of nelfinavir resulted in more profound radiosensitisation in PSN-1 xenografts when co-injected with PSCs (Al Assar et al., under review). Nelfinavir can decrease hypoxia and improve blood flow in xenograft and spontaneous mouse tumour models [10], [11]. In ARC II, similar changes in hypoxia and perfusion were demonstrated by FMISO-PET and pCT, although these were exploratory end-points. This is the first clinical study to demonstrate improved tumour oxygenation and perfusion using a biological agent in pancreatic cancer and suggests potential role of imaging biomarkers for patient selection in future trials.

The above preclinical and clinical findings on nelfinavir are important in the context of pancreatic cancer microenvironment. The hypovascularised immunosuppressive desmoplastic stroma mediates chemo- and radioresistance and promotes progression [8], [9], [38], [39], [40]. Hence, nelfinavir might constitute a promising agent to modify the tumour microenvironment towards a more physiological state to improve the clinical outcome after CRT as it is suggested by preclinical data (Al-Assar et al. submitted to Radiother Oncol).

Our study had several shortcomings. Although the results are promising, the benefits of nelfinavir over and above CRT cannot be ascertained, as this is not a randomised study. Secondly, the outcome from this trial needs to be interpreted with caution as it closed early. Thirdly, the predominance of systemic failure suggests that induction chemotherapy may have allowed better selection of patients for CRT, however the study was designed prior to reporting of SCALOP and LAP07 [24], [26]. Finally, the large radiation fields used in this study to encompass uninvolved regional lymph nodes may not be necessary [41], [42] and may have contributed to the high incidence of Grade 3–4 toxicity seen in this study. In future trials (including SCALOP2), the radiation volume should exclude prophylactic regional nodal irradiation.

In summary, notwithstanding these limitations, ARC II does demonstrate that CRT can be delivered safely with Nelfinavir, and the clinical outcome is promising. A 5-arm randomised phase II study, SCALOP2, will open shortly in the UK and other centres in Europe, where all patients will receive 3 months of Gemcitabine plus Nab-Paclitaxel chemotherapy followed by randomisation to continuing further chemotherapy, 50.4 Gy in 28 fractions Capecitabine-based CRT (with/without Nelfinavir) and 60 Gy in 30 fractions Capecitabine-based CRT (with/without Nelfinavir). SCALOP-2 trial will attempt to provide a more vigorous validation of these results.

## Grant support

We gratefully acknowledge the funding support of Cancer Research UK, Kidani Memorial Trust, Medical Research Council and the NIHR Biomedical Research Oxford.

## Disclosure of potential conflict of interest

No conflicts of interest in respect to this work for any of the authors.

SM is part funded by MRC (Medical Research Council) and NIHR Oxford Biomedical Research Centre.

This work was supported by the CRUK & EPSRC Cancer Imaging Centre Oxford [grant No. C5255/A1646].

JMW has received an educational grant from Astellas UK and is funded by CRUK & EPSRC Cancer Imaging Centre in Oxford grant No. C5255/A1646.

MAH is funded by the MRC grant MC_PC_12001/2.

MP is funded by CRUK (C5255/A15935).

No further disclosures.

## Figures and Tables

**Table 1:** Patient and tumour characteristics at baseline in the entire cohort (*n* = 23).

Age in years: mean (SD)	64.7 (7.27)
Sex: female: male [*n* (%)]	10 (43.5%): 13 (56.5%)
% Karnofsky performance status [*n* (%)]	
>80	15 (65.2%)
70–80	7 (30.5%)
Not documented	1 (4.3%)
Enrolment to start of treatment in days (median; IQR; range)	14; 9, 17; 4–23
Tumour diameter (mm) (mean; IQR; SD; range)	33.5; 28, 40; 9.24; 16–51
CA19-9 concentration U/ml (median, IQR)	387 (68,1711)

SD: standard deviation; IQR: inter quartile range.

**Table 2:** Adverse events of CTCAE (version 4) Grade 3–4.

Toxicity	Number of participants: *n* (%)
Any Grade 3–4 effects	23 (100%)

Haematological	13 (56.2%)
Haemoglobin	0 (0%)
Leucocytes	7 (30.4%)
Absolute neutrophil count	2 (8.7%)
Platelets	9 (39.1%)
Lymphocytes	20 (87.0%)

Non-haematological	23 (100%)
Constitutional symptoms	5 (21.7%)
Fatigue	4 (17.4%)
Weight loss	0 (0%)
Syncope	1 (4.3%)
Other	0 (0%)
Dermatological symptoms	0 (0%)
Gastrointestinal symptoms	8 (34.8%)
Diarrhoea	5 (21.7%)
Nausea or vomiting	5 (21.7%)
Anorexia	0 (0%)
Other	4 (17.4%)
Infection	3 (13.0%)
Sepsis	2 (8.7%)
Cholangitis	1 (4.3%)
Other	0 (0%)
Vascular	1 (4.3%)
Thrombosis, thrombus or embolism	1 (4.3%)
Other	0 (0%)
Metabolic (laboratory)	10 (43.5%)
Liver	7 (30.4%)
Other	5 (21.7%)
Other	2 (8.7%)

## Appendix A Supplementary data

Supplementary Table 1 Treatment compliance during treatment.
