# Supplementary material for: ARCII: A phase II trial of the HIV protease inhibitor Nelfinavir in combination with chemoradiation for locally advanced inoperable pancreatic cancer
Source: Radiother Oncol. 2016 May;119(2):306–11. doi: 10.1016/j.radonc.2016.03.021 (PMC4917892; doi:10.1016/j.radonc.2016.03.021)
Supplement: Supplementary Table 1 — Treatment compliance during treatment. [file mmc1.docx]

Supplementary Table 1. Treatment compliance during treatment

| **Percentage of protocol dose** | **Number of participants (%)** | | |
| --- | --- | --- | --- |
|  | **Gemcitabine** | **Cisplatin** | **Nelfinavir** |
| 100% | 14 (60.9%) | 14 (60.9%) | 12 (52.2%) |
| 80-99% | 4 (17.4%) | 4 (17.4%) | 8 (34.8%) |
| 50-79% | 3 (13.0%) | 3 (13.0%) | 0 (0%) |
| 1-49% | 0 (0%) | 0 (0%) | 3 (13.0%) |
| None | 2 (8.7%) | 2 (8.7%) | 0 (0%) |
| **Radiotherapy** | | | |
| **Total radiotherapy dose (Gy)** | **Number of participants (%)** | | |
| 59.4 | 17 (73.9) | | |
| >=54 | 20 (87.0%) | | |
| >=45 | 21 (91.3%) | | |
